# Svensson class IV Ascending aortic dissection, often confused with penetrating ulcer

**DOI:** 10.15171/jcvtr.2015.09

**Published:** 2015-03-29

**Authors:** Michel Francklyn Mitsomoy, Valerica Alexoiu, Matthias Kirsch

**Affiliations:** AP-HP, Hôpital Bichat Claude- Bernard, Department of Cardiac Surgery and Heart Transplantation, Paris, France

**Keywords:** Aortic Dissection, Svenson Classification, Aortic Ulcer

## Abstract

TWe present the case of a 64 years old male patient who had recently suffered an infective aortic valve endocarditis (*Streptococcus agalactiae*) complicated by embolic arthritis of the right hip. Initial echocardiography revealed moderate aortic insufficiency developed on a tricuspid aortic valve with a small vegetation (5 mm × 4 mm) on the left coronary cusp. Furthermore, an aneurysmal dilatation of the ascending aorta (maximal diameter, 54 mm) was noted. Other heart valves and left ventricular function were considered normal. The patient completed a 4 weeks course of antibiotherapy, and the right hip arthritis was treated by drainage and synovectomy. The patient was subsequently referred to surgery on an outpatient basis for the aneurysm of the ascending aorta. Preoperative computed tomography showed localized aortic dissection of the tubular ascending aorta characterized by an intimal tear without medial hematoma but excentric bulging of the aortic wall. This lesion was initially considered a penetrating ulcer of the aortic wall The operative specimen allowed to make differential diagnosis with a penetrating aortic ulcer by showing that the lesion did not develop within an atherosclerotic plaque. However, downstream extension of the dissection was probably limited by the presence of transmural calcifications on its distal side. The patient underwent successful complete aortic root replacement using a stentless Freestyle bioprosthesis with Dacron graft extension as reported previously.

## Introduction

In 1999, Svensson and colleagues proposed a pathophysiological classification for aortic dissection, which was subsequently adopted in medical practice.^1-3^ This classification distinguishes five subtypes, among which subtype IV, also known as discrete or localized aortic dissection, is of rare occurrence and remains poorly understood.^4^ In some cases, the subtype IV can be confused with penetrating ulcer.

## Case presentation

We present the case of a 64 years old male patient who had recently suffered an infective aortic valve endocarditis (*Streptococcus agalactiae*) complicated by embolic arthritis of the right hip. Initial echocardiography revealed moderate aortic insufficiency (2/4; regurgitant orifice area, 19 mm^2^; regurgitant volume, 59 mL) developed on a tricuspid aortic valve with a small vegetation (5 mm × 4 mm) on the left coronary cusp. Furthermore, an aneurysmal dilatation of the ascending aorta (maximal diameter, 54 mm) was noted. Other heart valves and left ventricular function (LVEF, 60%, LVEDD, 49 mm) were considered normal. The patient completed a 4 weeks course of antibiotherapy and the right hip arthritis was treated by drainage and synovectomy. The patient was subsequently referred to surgery on an outpatient basis for the aneurysm of the ascending aorta.

**Figure 1:**
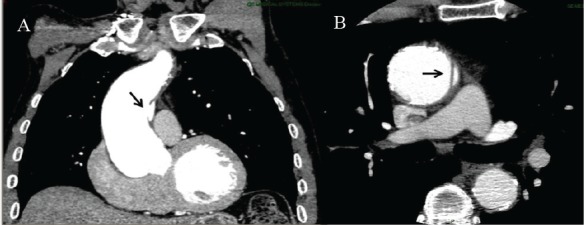
Preoperative computed tomography scan: (A), sagittal view; (B), axial view. Note the intimal tear without medial hemaoma and eccentric aortic wall bulge (arrows).

**Figure 2:**
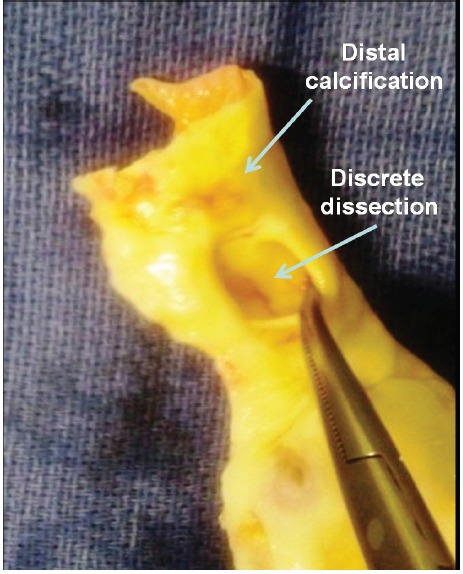
Operative specimen of the tubular ascending aorta. Note the localized dissection, limited on its distal side by aortic calcifications.

Preoperative computed tomography showed localized aortic dissection of the tubular ascending aorta characterized by an intimal tear without medial hematoma but excentric bulging of the aortic wall (This lesion was initially considered a penetrating ulcer of the aortic wall) (Figure 1). The operative specimen allowed to make differential diagnosis with a penetrating aortic ulcer by showing that the lesion did not develop within an atherosclerotic plaque. However, downstream extension of the dissection was probably limited by the presence of transmural calcifications on its distal side (Figure 2).

## Discussion

There are two well-recognized forms of aortic dissection: dissection of the aortic wall, resulting in the classic septum associated with an intimal tear (class 1), and the less common intramural hematoma-type dissection of the aortic wall in which the dissection is usually filled with blood clot without a detectable intimal tear (class 2).^6,7^ In patients with a classic intimal flap, detection of the presence of aortic dissection by available imaging techniques is very accurate, with a reported sensitivity of 97% to 100% for both transesophageal echo (TEE) and MRI.^6,7^

The importance of variants such as intramural hematoma (class 2) dissections and penetrating ulcer (class 4) sometimes present some diagnostic difficulties.

For patients with an intramural hematoma-type dissection, the sensitivity for detection of dissection is difficult to document accurately because it is not known how many patients are missed (false negatives). Furthermore, noninvasive and invasive testing may overestimate the incidence of this type of dissection because a tear is often found at the time of surgery or autopsy.^6^

The variant class 3 of aortic dissection is characterized by a stellate or linear intimal tear associated with exposure of the underlying aortic media or adventitial layers but without the progression and separation of the medial layers, but only extensive undermining of the intimal layers. And the class 5 is about iatrogenic or traumatic aortic dissection.

## Conclusion

The patient underwent successful complete aortic root replacement using a stentless Freestyle bioprosthesis (Medtronic MN, USA) with Dacron graft extension as reported previously.^5^

## Ethical issues

The authors have obtained all permission before using any data and patient images.

## Competing interests

Authors declare no conflict of interests in this study.
